# Skin Diseases and Syphilis

**Published:** 1894-01

**Authors:** 


### *SELECTIONS AND ABSTRACTS.*

### SKIN DISEASES AND SYPHILIS.

EDITED BY DR. M. B. HUTCHINS.

### URTICARIA AND DEATH AFTER AN OVARIOTOMY.

(*Centralb. für Gynekol.*) Omori and Ikeda (Japan). On the fifth day after the operation the temperature (of the fifty-four-year-old patient) suddenly rose. The whole body was covered with an urticarial eruption. Peritonitic symptoms appeared and death occurred in a few hours.—*Monatsh. für Pr. Derm., xvii., No. 11.*

(Another of the many things which may excite an urticarial eruption.)

### LOCAL TREATMENT OF BURNS.

Faytt (*Gaz. Lek.*, 1893, No. 6, from *Centralb. für Chirurg.*, No. 36) first uses cloths wet with "salicylic bichloride solution," or "liq. Goulardi," until the necrotic tissue can be removed. Then he covers the wound with a large piece of protective silk, covering this with an antiseptic dressing. The merit of this plan lies in the fact that the secretion easily finds its way along the smooth under surface of the silk to the edge, where it is taken up, the dressing does not stick, pain limited and there is less tendency to contraction of the scars.—*Mon. für P. Derm., xvii., No. 11.*

### BURNS.

*Monatshefte für Prak. Dermatologie* has a reference to a recommendation of Capitan for the treatment of these injuries. Cleanse with boric acid water or ½ per cent. bichloride solution, cauterize ulcerous points with the red-hot needle, and the application of following salve (aseptic hands): salol 4 parts, cocaine mur. ½ of 1 part, vaseline 30 parts, and over this a wet, bichloride dressing, this to be kept wet. Every two or three days, change of dressing. Claims that this treatment prevents pus and pain.

---

### Lupus from Inoculation.

Gram (*Bruns. Beitr. z. Klin. Chirurg.*) reports four such cases. Referring to different tubercular skin lesions resulting from like cause asks the reason. He mentions three possibilities; differing virulence of the tubercle bacilli; varying powers of resistance in individuals, and difference in point of infection, as not only wounds but the hair follicles and mouths of sebaceous glands may serve as a point of entrance.—*M. für Prak. Derm., xvii., No. 11.*

---

### Condylomata Lata.

At the clinic for skin diseases and syphilis in the Atlanta Medical College recently a marked case of this appeared. There was the scar from a preputial chancre, the adenopathy, mucous patches over pillars of pharynx and history of a general eruption. On each side of anus a half-dollar sized warty growth; flat, papular, covered with a mucoid discharge. Lesions extremely sensitive. "Mixed treatment," with calomel to the condylomata. Patient, a disreputable negro boy seventeen years old.

---

### The Etiology of Cancer.

Professor Duplay and M. Cazin discuss in the *Semaine Médicale* some of the more recent assertions made in various quarters on the etiology of cancer. In connection with the possibility of a more or less direct transmissibility of cancer from one member of a household to another, or among the inhabitants of a locality, the evidence is very meagre. Arnaudet asserted the frequency of cancer in the Canton of Cormilles, where it exists in somewhat epidemic form and localized to certain districts. Arnaudet suggested that the disease was conveyed through drinking water, and especially cider made with contaminated water, or by defective sanitation of dwellings. Professor Brono's subsequent investigations, however, failed to afford any corroboration. Feissenger traced four cases of cancer in three houses to contamination, apparently with the dressings from a case of scirrhus of the breast. Guelliott and Fabre collected instances of "house-epidemics." In this connection Metchnikoff's hypothesis must be borne in mind that cancerous neoplasus might possibly belong to the group of miasmatic diseases capable of being propagated by spores formed outside the organism.

It has long been known that cancer is easily disseminated throughout the organism by cancerous particles detached from the primary growth and deposited in the various organs and tissues. Secondly, experiments on the human subject have shown that the inoculation of cancer to patients already suffering from the disease is not necessarily followed by positive results, though some have been observed.

Several investigators have recently claimed to have successfully inoculated human cancer to rats and mice, but a long series of prior attempts were apparently unsuccessful, and the more recent ones are open to doubt. In the inoculation from one animal to another of the same species negative results were obtained by numerous investigators, but on the other hand a few positive ones have been arrived at, viz., by Goujon (epithelioma in guinea-pig), Klencke (melanotic carcinoma in the horse), Wehr (carcinoma in the dog), Hanau (epithelioma in the rat), Eiselsberg (filio-sarcoma in rat), and Morau (epithelioma in mice). Hanau's well-known results are very striking.

Quite recently Morau has presented to the Academy of Sciences the following conclusions arrived at in his continued experiments.

1. White mouse epithelioma is inoculable from one animal to another of the same species.

2. Hereditary influences play a very important part in the development of these tumors.

3. Secondary growths are apt to form in various parts of the body, the development of these metastatic formations being favored by an injury to the tissues or organs.

4. Gestation produces the same effect as traumatism.

5. Certain toxines are produced in the substance of these tumors. They are absorbed into the system giving rise to general cachexia.

6. These tumors apparently lose their virulence by successive transmission through a series of animals of the same species.

7. So long as the skin is unbroken these tumors are apparently free from bacteria.

8. Picric acid seems to exert a favorable influence on these tumors by coagulating the protoplasm of the cells. For this purpose it must be brought directly in contact with the pathogenic cells (interstitial injections).—*British Journal of Dermatology, November, 1893.*

---

HYDROA VACCINIFORME, BAZIN. DR. C. BOECK. (*Norsk Magazin for Lægevidenskaben, No. 6, 1893.*)

The writer reports three cases of this rare skin affection, which has been brought to the attention of dermatologists by Jonathan Hutchinson after being first described by Bazin. In the first the patient, a boy nine years, had suffered for three consecutive summers from vesicles and deep bullæ, from the size of a pin point to that of a pea, disseminated symmetrically over the cheeks, the ear, with traces on the forehead, back and right hand. On the ears especially the vesicles were large and deep, with slight hemorrhage into the base of each. After having lasted for several days they became depressed in the center and a brownish crust began to form, ending by covering the whole of the bulla, which, after falling off, left a cicatrix on the skin, often of great depth. Violent itching accompanied the eruption, which this time appeared in May. In the second case, a three year old boy, it appeared, for the third summer, symmetrically on the nose and cheeks, the back of the hands and the internal side of the forearm. The vesicles were as large as a bean; drying, they formed crusts which, on falling off, left deep cicatrices. The third spring the eruption developed at the first time that he ventured out. This time his ears were also attacked. At the base of each bulla, which were firm, hard and translucent violet-colored points, as in certain cases of acne necrotica, were seen. The third case, a young woman of twenty-seven years, the disease had been observed for the first time the preceding spring, especially upon the chin and ears, as well as slightly on the extremities and trunk. In this case the eruption would appear after a walk in the open air and even a short promenade. The cicatrices were not as deep as in the other two cases. The process occurring in the skin is similar to that in acne necrotica.—*Journal Cut. and Gen. Ur. Dis., December, 1893.*

---

HERPES SEU DERMATITIS GESTATIONIS. DR. BOECK. (*Norsk Magazin for Lægevidenskaben, No. 6, 1893.*)

The author describes a case in a woman of twenty-eight years, in whom, two days after her second confinement, an eruption appeared on the palms of her hands and the soles of her feet, accompanied by violent itching. During her third pregnancy, when he observed her, it set in at the fifth month and lasted for eight weeks after labor, consisting then of vesicles, large bullæ, papules and erythematous plaques. It was absolutely symmetrical and affected all four extremities and the trunk. During the fourth pregnancy it appeared at the fourth month, by a slight eruption on the forearms. During the fifth pregnancy reappeared at the third month and persisted for four or five months after labor. The attack this time was more violent and painful, being accompanied by pustules.—*Journal Cut. and Gen. Ur. Dis., December, 1893.*

---

DERMATITIS HERPETIFORMIS CIRCUMSCRIPTA SEU CIRCINATE. DR. C. BOECK. (*Norsk Magazin for Lægevidenskaben, No. 6, 1893.*)

The writer records two cases of a type as yet unmentioned. In the first, a woman of thirty years, on the dorsal surface of each hand there was a circular plaque of the size of a child's hand, with the central portion infiltrated and hyperemic, and this surrounded by a peripheric portion of small translucent vesicles. Similar patches of the size of a hazelnut were on the first phalanges of the fingers, and also were surrounded by small vesicles. Intense itching. It first appeared six years before and had reappeared each winter. In the second case, a man of twenty-three years, the affection was less characteristic; the plaques were very similiar and had developed each winter for two winters, especially on the right hand.—*Journal Cut. and Gen. Ur. Dis., December, 1893.*

